# Health Communication Research Informs Inflammatory Bowel Disease Practice and Research: A Narrative Review

**DOI:** 10.1093/crocol/otad021

**Published:** 2023-04-21

**Authors:** Neda Karimi, Alison Rotha Moore, Annabelle Lukin, Susan J Connor

**Affiliations:** South Western Sydney Inflammatory Bowel Disease Research Group, Ingham Institute for Applied Medical Research, Sydney, New South Wales, Australia; School of Clinical Medicine, Faculty of Medicine and Health, The University of New South Wales, Sydney, New South Wales, Australia; School of Humanities and Social Inquiry, Faculty of Law, Humanities and the Arts, The University of Wollongong, Wollongong, New South Wales, Australia; Department of Linguistics, Faculty of Medicine, Health and Human Sciences, Macquarie University, Sydney, New South Wales, Australia; South Western Sydney Inflammatory Bowel Disease Research Group, Ingham Institute for Applied Medical Research, Sydney, New South Wales, Australia; School of Clinical Medicine, Faculty of Medicine and Health, The University of New South Wales, Sydney, New South Wales, Australia; Department of Gastroenterology, Liverpool Hospital, Sydney, New South Wales, Australia

**Keywords:** clinical communication, doctor–patient relationship, patient involvement, disease outcomes, telehealth

## Abstract

**Background:**

In the absence of targeted empirical evidence on effective clinical communication in inflammatory bowel disease (IBD), a broad overview of existing evidence on effective communication in healthcare and available recommendations for communication in telehealth is provided and mapped onto IBD research and practice.

**Methods:**

A narrative literature review was conducted using Pubmed and Scopus databases and snowballing literature search.

**Results:**

Evidence-based *relationship building* strategies include communicating emotions, acknowledging and addressing patients’ hesitancy, and ensuring continued support. A particular recommendation regarding telehealth interaction is to avoid long stretches of talk. Effective *informational* strategies include facilitating and supporting information exchange and considering patients’ preferences in decision-making. In teleconsultations, clinicians should ask direct questions about patients’ emotional state, clarify their understanding of patients’ concerns and check patients’ understanding, address at least one patient-reported outcome when discussing the recommended treatment, and shorten the consultation where possible. Strategies for maximizing effective clinical communication in the spoken *communicative mode* include using infographics and simple language, and assessing adherence at the beginning of the consultation. For teleconsultations, clinicians are advised to allow patients to explain the reason for their call at the beginning of the teleconsultation, probe additional concerns early and before ending the teleconsultation, and be mindful of technical issues such as voice delays.

**Conclusions:**

Use of question prompt lists, decision aids, micro-lessons, and communication training interventions for clinicians could be beneficial in IBD care. Further research into the implementation of such interventions as well as clinical communication concerns specific to IBD is warranted.

## Introduction

Effective clinical communication is crucial for optimal patient care in inflammatory bowel disease (IBD),^1^ a chronic inflammatory condition of the gastrointestinal tract characterized by intermittent periods of active disease with symptoms that undermine patients’ quality of life and emotional well-being. Yet research evidence on how communication strategies can improve clinical outcomes in IBD is scarce.

In Australia, a 2016 national audit of the provision and organization of IBD services revealed a significant disease burden which could be alleviated by appropriate and well-coordinated care.^2^ Audit recommendations repeatedly involved the relationship between patients and their IBD team, indicating the important role of clinician–patient communication in reducing this disease burden. A related survey of over 700 IBD patients in Australia^3,4^ that came out of the audit confirmed many of the audit’s findings from the patient’s point of view and highlighted some additional concerns relevant to communication. In particular, 51% of the respondents wanted to be more involved in decisions regarding their care.^3^ Half of all respondents registered as mildly, moderately, or severely distressed on psychological testing but only 32% of those in severe distress were receiving care from a mental health clinician.^4^ Correspondingly, 56% of the patients who were not asked about their mental health by their specialist or IBD nurse wanted to be asked about this. Many patients also reported in this survey that no contingency plans were in place, or had never been discussed with their IBD clinicians.^3^

Internationally, the small set of studies we have resonates with the Australian picture, indicating that communication between gastroenterologists and patients has an important role in addressing clinical concerns around improving disease management and patient self-management, and avoiding unnecessary hospitalizations.^5–11^ These results regarding the role of communication are consistent with reported patient priorities internationally, including wanting more information earlier in their disease course^12^ and wanting to discuss the effect of IBD on personal relations and the patient’s quality of life during consultations.^7^ The latter could also help explain why patients turn to sources outside conventional medicine for information and treatment of IBD.^13–15^ These results fit with our understanding beyond IBD that most complaints about doctors are made about communication, not clinical competencies.^16–18^

Existing research on how to address these concerns is limited. Targeted empirical evidence on effective clinical communication in specific types of healthcare encounters is key for making conscious and sustained changes.

In the absence of evidence specific to IBD,^19,20^ this narrative review provides an overview of existing evidence on effective clinical communication from the broader health communication literature. We use the results to consider and prioritize existing gaps in research and practice in IBD. Given the recent turn to telehealth visits in IBD and the likelihood that this practice will continue, we particularly wanted to include knowledge on effective communication in telehealth settings, even though the literature on this has a smaller empirical base. Thus, we sought answers to the following review questions:

- What aspects of clinical communication have been shown to be associated with improved disease outcomes in chronic diseases?
- What evidence- or consensus-based recommendations are available on clinical communication in telehealth?
- How can this knowledge help identify priority points of communication intervention in IBD?

## Methodology

A literature search was performed using Pubmed and Scopus databases from inception through December 2022. The following keywords were used to identify relevant publications in English through title and abstract screening first and full-text review subsequently:

“chronic,” “communication,” “doctor-patient,” “clinician-patient,” “disease outcomes,” “patient-reported outcomes,” “telehealth,” “telephone consultation,” “video consultation,” and “telemedicine.”

Reference lists of selected articles as well as subsequent citing publications of landmark articles were screened for additional relevant articles.

Quantitative and mixed-methods studies that investigated associations between aspects of clinical communication and health outcomes or the effect of clinical communication on outcomes in chronic disease management were considered eligible. In addition, publications on telehealth were included if they made recommendations on clinician–patient communication in their results or conclusion sections regardless of study design. This looser selection criterion for telehealth was set because of limited evidence in this area despite increasing research on communication in telehealth since the COVID-19 pandemic began.

Data extraction and synthesis were performed in Excel and NVivo.

## Results and Discussion

The bulk of research on communication between healthcare providers and healthcare consumers focuses on primary care, oncology, and end-of-life care. Studies that investigate communication in chronic disease management (excluding cancer care) constitute a considerably smaller share of the literature. Mapping this research to the IBD setting requires care but is a useful starting point for considering what we already know and where priorities lie.

Results from empirical studies on the relation between communication style and health outcomes in chronic disease management are presented followed by a summary of work on clinical communication in telehealth. Due to limited availability of empirical work on communication in telehealth, best-practice recommendations as well as any available findings are included.

**Figure 1.**
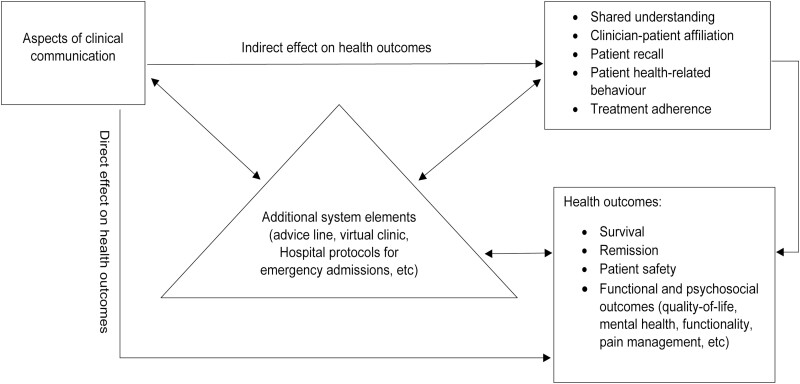
Clinical communication and health outcomes.

Tracking the effect of communication styles all the way to clinical outcomes is very difficult. This is because communication is more likely to affect health in an indirect way through its influence on intervening variables^21–24^ such as shared understanding,^25^ patient recall,^26,27^ patient health-related behavior,^27,28^ and patient adherence^29–33^ (Figure 1).

Nevertheless, there has been a moderate amount of research relating clinical outcomes to the nature of interaction between patients and clinicians over the past 40 years.

Available evidence is classified and synthesized around the following priorities outlined in the Australian IBD audit:

- Patient involvement, defined as patients being actively involved in decisions about their care, and
- Psychological support, defined as support provided by clinicians to help meet the mental, emotional, social, and spiritual needs of patients and their families.^34^

Table 1 provides the included publications and a summary of the available evidence on communication strategies that are linked to improved outcomes in chronic disease management.

**Table 1.** Characteristics of included studies linking clinical communication to improved outcomes in chronic disease management.

	Year	Design	No. of participants	Disease	Communication strategies (patient)	Communication strategies (clinician)
^31^	2017	Interventional cohort study	Patients (*n* = 299); healthcare providers (*n* = 13)	HIV	Not studied	•Assessing and empowering adherence every 3–6 months •Setting the agenda at the beginning of the consultation **For patients with adherence issues:** 1.Showing empathy and acknowledging that adherence requires effort and can sometimes be challenging 2.Assessing adherence and reasons for nonadherence 3.Assessing and orienting to stage of readiness: •*Precontemplation* Show respect/Understand underlying beliefs/Establish trust/Provide short, individualized information/Schedule next appointment •*Contemplation* Allow ambivalence/Support weighing pros and cons with the patient/Assess information needs and support information seeking/Schedule next appointment •*Preparation* Reinforce decision/Use shared decision-making (SDM)/Educate (adherence, resistance, side-effects, discuss integration in daily life, assess self-efficacy)
^35^	1987	Correlational	Patients (*n* = 170); physicians (*n* = 9); physician assistants (*n* = 2)	Hypertension	Disclosure or clarification of information about history and symptoms	Providing objective information about illness and treatment
^36^	1989	Correlational (data from experimental studies)	Patients (*n* = 252)	Ulcers, hypertension, diabetes, breast cancer	•Controlling behaviors: Asking questions, attempts to direct the flow of conversation and physician’s behavior (interruptions, suggestions, assertations) •Expression of affect (emotion) (negative and positive) •Effectiveness in obtaining information: the ratio of factual statements by the physician to controlling behaviors by the patient •Involvement: the ratio of patient to physician conversational behaviors	•Information provision and exchange of opinion and affect (emotion)
^37^	2009	Experimental (cluster randomized controlled trial [CRCT])	Patients (*n* = 417)	Type 2 diabetes	Involvement: requesting the doctor to address essential aspects of their care	Not studied
^38^	2010	Correlational (path analysis)	Patients (*n* = 166)	Type 2 diabetes	Patient activation: •Preparing a list of questions for the doctor •Asking questions about treatment •Discussing personal problems related to illness	Physician participatory decision-making: •Offering choices in medical care •Discussing pros and cons of each choice with the patient •Asking about patient’s preferences •Taking patient’s preferences into account when making treatment decisions
^39^	2003	Correlational	Patients (*n* = 79); rheumatologists (*n* = 7)	Systemic lupus erythematosis (SLE)	Patient participation: question asking, assertive responses, and expressions of concern	No significant correlations found
^40^	2014	Correlational	Patients (*n* = 135); physicians (*n* = 24)	Advanced cancer	Asking questions, expressing concerns, and stating preferences	Not studied
^41^	2006	Experimental	Patients (*n* = 195); physicians (*n* = 11); nurses (*n* = 26); physiotherapist (*n* = 1)	Heart surgery	Not studied	•Information provision •Supporting patient involvement and co-production •Standardizing communication with patients
^42^	2017	Correlational	Patients (*n* = 6810)	Atherosclerotic cardiovascular disease	Not studied	•Explaining things in a way that is easy to understand •Showing respect for what patients has to say •Spending enough time with the patient •Effective listening
^43^	2008	Experimental (CRCT)	Patients (*n* = 985)	Hypertension, systolic blood pressure	Not studied	Communication of absolute and potentially modifiable risk of negative outcome and education on modifiable risk factors and their control through behavioral changes and drug therapy
^44^	2021	Single group pretest–posttest longitudinal pilot	Patients (*n* = 50)	HIV	Not studied	Use of infographics in patient education especially in patients with low health literacy
^45^	2021	Experimental	Patients (*n* = 80)	Asthma	Not studied	•Raising the subject of nonadherence and disease control •Making an explicit connection between nonadherence and uncontrolled disease •Enhancing motivation to increase adherence by exploring and resolving ambivalence toward treatment •Engaging in SDM
^46^	2003	Correlational	Patients (*n* = 74); physician (*n* = 38)	Diabetes	Not studied	•Assessing the patient’s recall and understanding by explicitly asking the patient to restate a new piece of health information or advice (including a significant change in management plan) •Eliciting the patient’s perceptions regarding a new piece of health information or advice (including a significant change in management plan)
^47^	2018	Randomized experiment	Analogue patients (*n* = 60)	Cancer	Not studied	Supportive communication: •Promise of continued support and best care possible •Acknowledging the patient’s hesitancy to accept the doctor’s recommendation in relation to a major health decision •Ensuring that all the patient’s concerns are addressed
^48^	2011	Correlational	Patients (*n* = 891); family physicians (*n* = 29)	Diabetes	Not studied	Empathy: an understanding (rather than feeling) of pain and suffering of the patient, combined with a capacity to communicate this understanding, and an intention to help

### Patient Involvement

The following communicative behaviors by the patient have been shown to be linked to or result in improved disease control biomarkers; better health; fewer health issues, days off work, and functional limitations due to illness; better pain management; and lower index of symptom experience^35–40^:

- describing their illnesses and circumstances in their own words,
- attempts at asking questions and directing the flow of conversation,
- expressing questions, concerns, assertive responses, and statements of preferences,
- requests for clinicians to address essential aspects of care, and
- effectiveness in obtaining information (the ratio of factual statements by the physician to the patient’s attempts at asking questions and directing the flow of conversation).

These finding are relevant in IBD where disease control, symptom management, quality of life, and healthcare utilization are important and where patient involvement should be a priority.^2^ As such the findings should be used as a starting point for IBD service providers, clinicians, and researchers in their effort to promote patient involvement. Priority should be placed on the development and implementation of user-friendly consumer interventions that encourage an active patient role. One useful type of intervention to achieve this is the question prompt list which is a structured list of potential questions that a patient may want to ask their healthcare provider.^49^ In addition, consumer interventions that provide education on the different aspect of care, including relationships with the IBD service clinical team, can support patient involvement. Examples of such interventions include patient decision aids and micro-lessons. IBD patient decision aids^50–52^ not only provide information on the different treatment options for IBD but also promote a more agentive role for the patient. Micro-lessons are short recorded audio or video presentations on a single, tightly defined topic and have been shown to engage learners and reduce mental fatigue.^53^ An example of an IBD-specific micro-lesson is the Healthline IBD micro-lesson, the clinical usefulness of which requires further research.

Looking at the physician’s communicative behaviors, more control of communication flow by the physician in the form of asking questions, giving directions, and interruptions has been shown to be related to poorer reported health.^38^ By contrast, the following behaviors have been shown to be linked to or result in improved health and lower risk of illness, better utilization of evidence-based therapies, and reduced costs^31,36,38,41–46^:

- provision of information, in general, and provision of information regarding modifiable risk factors,
- support of patient co-production of clinical interventions,
- support of shared decision-making (offering choices in medical care, discussing pros and cons of each choice with the patient, asking about patient’s preferences, and taking them into account in decision-making),
- providing medical information in simple language,
- asking the patient to restate information or instructions to avoid misunderstanding, especially in patients with lower health literacy,
- use of infographics during routine medical encounters—especially in patients with low health literacy, and
- involving the patient in addressing adherence issues (setting the agenda at the beginning of the consultation, assessing adherence and reasons for nonadherence, showing empathy, eliciting the patient’s beliefs, supporting information seeking, shared decision-making, and considering the patient’s individual circumstances).

Targeted research on the way doctors and patients discuss costs and benefits of medications is likely to be useful in IBD where medication take-up and adherence is an important clinical focus, along with lifestyle measures, over many phases of life.^54^ In addition, clinician training interventions to explore and address adherence issues in IBD should be a research priority. In such training, nonadherence should be regarded as a dynamic and relational factor that can happen from time to time for various reasons, for any patient.^55,56^ Beyond adherence alone, clinician training interventions with a broad focus on providing appropriate information and shared decision-making should be on the IBD research and practice agenda. Further, the use of the teach-back technique, a technique for verifying patients’ understanding of their health information,^57^ and information provision support interventions such as infographics will likely be helpful in IBD. One reason why such approaches assist is because limited health literacy is associated with poorer outcomes in IBD^58^ and complex medical concepts could create a communication barrier between patients and IBD clinicians and hinder patient involvement.

### Psychological Support

Standard mental health screening questionnaires that provide a quick snapshot of the patient’s mental health with the aim of identifying patients who need specialist psychological support are important and efficient tools for providing psychological support to patients with chronic diseases such as IBD. However, it is highly likely that patients who are assessed as not needing specialist psychological support may still benefit from some sort of psychological support from their treating clinician. For example, evidence shows that using supportive communication when delivering bad news lowers heart rate variability.^47^ Clinician behaviors known to provide support for the patient include stating one’s continued support and care, and acknowledging and addressing the patient’s concerns.^47^ In addition, empathy has been shown to be associated with positive clinical outcomes in diabetes.^48^ Findings from these studies are relevant to IBD where disease burden is high for patients and patients can be repeatedly confronted with bad news. Basic psychological support training for IBD clinicians including training on supportive communication strategies and empathic communication, therefore, should be a priority.

### Communication in Telehealth

Research on the use of e-health technology in IBD has been accumulating over the last 10 years, motivated in particular by the growing demands on limited IBD care resources, the complex nature of IBD, and the necessity of taking a “proactive” rather than “reactive” approach to disease management.^59,60^ The role of telehealth and remote IBD care in maintaining equitable and continuous care has been further reinforced by the COVID-19 pandemic.^61–63^ An important aspect of telehealth in IBD that has received little attention is clinical communication. Guidelines and telehealth communication competencies training that extends beyond technical challenges are lacking. Evidence on clinical communication in telehealth beyond IBD is also limited but has been growing since 2020 due to an acceleration in using and refining telehealth technologies during the pandemic.

The following recommendations have been extracted from health communication research beyond IBD care. Table 2 provides an overview of the selected literature for this narrative review.

**Table 2.** Characteristics of included studies on communication in telehealth.

	Year	Country	Context	Modality	Participants	Design
^64^	1997	Denmark	Emergency	Telephone	Doctors (*n* = 152)	Qualitative: Educational intervention development and role-play and debrief
^65^	2012	Sweden	Adviceline (malpractice claims)	Telephone	Patients (*n* = 33)	Mixed-methods
^66^	2021	Israel, Italy, Germany, Switzerland, United States, and Canada	Integrative oncology	Online	Integrative oncology experts (*n* = 54)	Consensus process
^67^	2021	United States	Surgical clinic	Video	Fourth-year medical students (*n* = 5); observers (*n* = 4)	Qualitative: Educational intervention development and role-play and debrief
^68^	2022	Australia	Gastrointestinal specialist clinic	Telephone	Doctors (*n* = 3) Patients (*n* = 14)	Qualitative: Conversation analysis
^69^	2020	United Kingdom	Diabetes service, antenatal diabetes service, hepatobiliary and pancreatic cancer surgery service, and heart failure service	Video and face-to-face	Patients (*n* = 65)	Qualitative: Ethnographic and conversation analyses
^70^	2021	Canada		Video and face-to-face	Healthy participants (*n* = 27)	Experimental
^71^	2019	Scotland	General practice	Video, telephone, and face-to-face	Patients (*n* = 149)	Quasi-experimental
^72^	2003	Canada	Adviceline	Telephone	Patients (*n* = 4696)	Correlational
^73^	2010	Scotland	General practice	Telephone and face-to-face	General practitioners (*n* = 18) Patients (*n* = 123)	Qualitative: Conversation analysis

#### Respect and Support the Patient Voice and Their Involvement in Telehealth

To respect the patient’s voice, it is recommended that patients are provided some time and space to explain the reason for their call as the first few seconds of a telehealth consultation are crucial and can influence how the consultation unfolds.^64^ This is particularly relevant to clinical communication in advicelines where patients are more likely to contact the adviceline with an urgent concern and where research has identified not adequately listening to the caller as the most common reason for malpractice claims.^65^ For both the clinician and the patient, telehealth is a healthcare setting with a risk of multiple distractions, thus any potential care challenges, including but not limited to concerns around medications (side-effects, safety, administration, etc.), the patient’s expectations of the treatment plan, communication issues, administrative issues, etc. should also be discussed or at least listed for discussion at the beginning of the telehealth consultation.^66^ This consensus-based recommendation was initially made in the context of integrative oncology, but we believe that the recommendation to discuss care challenges at the beginning of the consultation could apply to IBD telehealth consultations with specialists, nurses, and dietitians due to a higher risk of distraction associated with telehealth consultations, in general.

In telehealth consultations, it is recommended that clinicians ask direct questions about patients’ emotional state because a patient’s distress can be difficult to interpret in the absence of physical contact.^67^ To ensure understanding of the patient’s concerns, clinicians are advised to clarify their understanding of the patient’s concerns by providing a summary of the patient’s report and seeking their confirmation.^64^ It is also recommended that any potential additional concerns or questions are probed near the start of the consultation and, again, before ending.^64,68^ Clinicians are advised to address at least one patient-reported outcome in the discussion of the recommended treatment and encouraged to use an interactive approach to support patient involvement and avoid long stretches of unidirectional speech (monologue).^66^ While the general evidence on patient involvement that was discussed in the previous section still applies to communication in telehealth, clinicians should be mindful of limitations associated with this medium of communication including latency (voice delay), turn-taking, and overlapping talk that could potentially influence the dynamics of the consultation or create misunderstandings, especially for the patients.^69^

#### Clear and Concise Communication in Telehealth

Research has revealed a significant memory deficit for health information provided through telehealth compared with face-to-face consultations.^70^ This is a significant issue for those with comprehension difficulties including older patients with impaired speech perception, patients with cognitive-linguistic impairment, or those from culturally and linguistically diverse backgrounds. As such, it is recommended that clinicians consider shortening the telehealth consultation session by using high-quality supplementary resources that could be shared with the patients before, during, or after the session, if appropriate^66^ or consider discussing complex issues in a face-to-face consultation.^73^ Evidence from general practice suggests that telephone consultations are most appropriate for discussing single non-complex concerns and that discussions of new acute problems over the phone are almost always followed up by an in-person consultation.^73^ Shortening the telehealth sessions could also reduce telehealth-associated clinician cognitive load and result in enhanced clinician empathy.^74^ Clinicians are also advised to avoid using overly technical language when giving patients instructions regarding their care.^66,71^ Clinicians should ensure the information and instructions they give are clear to the patient by checking the patient’s understanding.^64,66,72^ Furthermore, allowing the patient to record the session and providing a written summary of the recommendations are among strategies recommended in the literature for effective communication in a telehealth setting.^66^

## Conclusion

**Figure 2.**
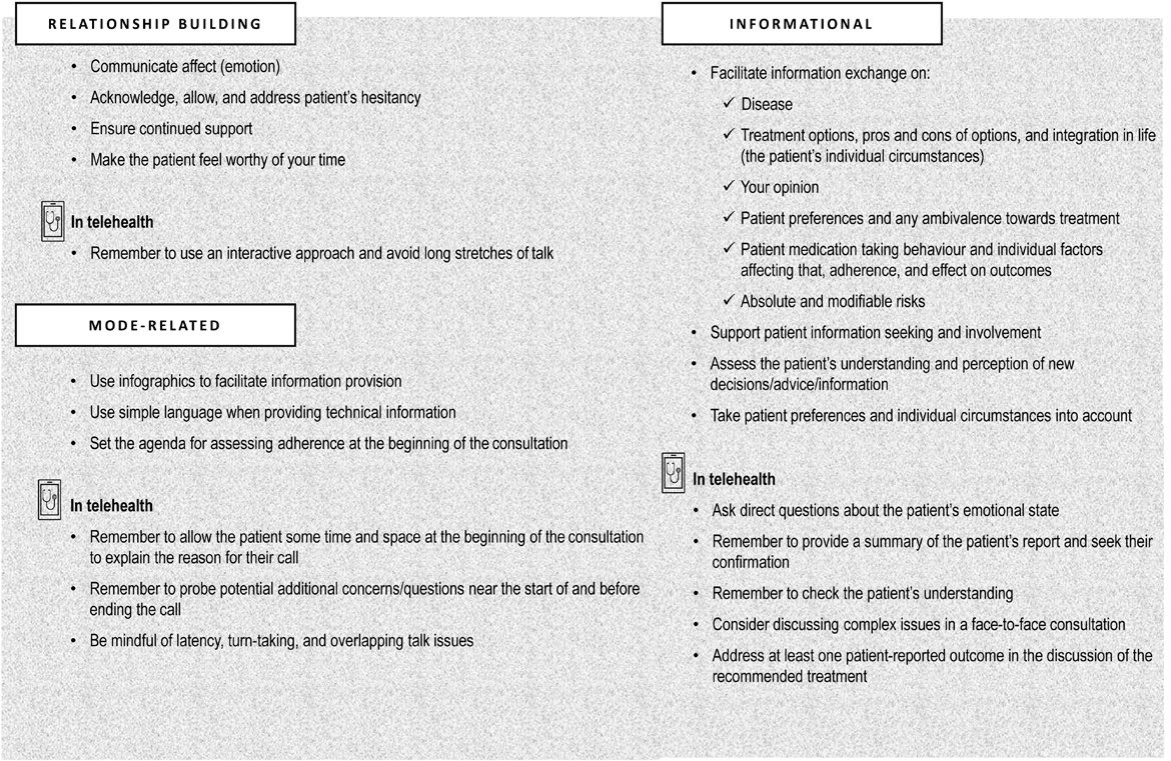
Effective strategies in communication with patients, by function.

In this narrative review, we reviewed the existing evidence on communication strategies that are linked to improved health outcomes in chronic disease management and have particular relevance for managing IBD. In addition, we synthesized the available recommendations for communication using telehealth technologies that are relevant to IBD. Figure 2 summarizes and classifies these communication strategies and recommendations in terms of the highly generalized function they serve: relationship building, informational, and mode-related. As their names suggest, relationship building strategies and informational strategies are aimed at building interpersonal relationships with the patient and exchanging information, respectively. Mode-related strategies concern making choices that suit the mode of IBD consultations in order to maximize effective care. These include choices that suit a predominantly spoken mode of communication, rather than the written mode as well as more specific choices depending on whether the consultation is in face-to-face or telehealth mode. Such strategies include changing the structure of talk, using simple language, or using visual aids. The representation of these strategies in Figure 2 can be used by clinicians as a quick visual guide while they are talking to their patients.

It also is important for future IBD research to engage with this evidence base in developing consumer communication support interventions as well as interventions for clinicians. In line with the synthesized evidence, the following communication support interventions could be beneficial in IBD management in terms of promoting patient involvement and improving disease outcomes:

- the question prompt list which is a structured list of potential questions that a patient may want to ask their healthcare provider,
- consumer interventions such as patient decision aids and online micro-lessons that focus on IBD, symptoms and their effect on daily life, medications, their usage and side-effects, healthcare maintenance, investigations and interpretation of results, as well as the relationship with the IBD clinical team including the patient’s role in IBD management and decision-making,
- clinician training interventions with a focus on information provision, shared decision-making exploring and respecting the patient’s voice and their values, and empathic communication,
- clinician training interventions for exploring and addressing adherence issues in IBD, and
- information provision interventions such as infographics to enhance patient understanding of complex medical concepts especially in patients with limited health literacy.

First, however, further research into these kinds of interventions and their implementation is needed. To take just one example, good infographic design relies on complex relations between verbal and graphic literacy among consumers and the judicious selection of clinical details and clinical metaphors. Although good infographics have the potential to make explanations clearer, poor design can in fact reduce accuracy of patient understanding.^75,76^

This narrative review of evidence from the generalist healthcare communication research is useful for thinking through what is most needed in IBD and how to address these needs but more IBD-specific research is needed. Communication research based on observation of how people with IBD talk with their doctors, nurses, pharmacists, dietitians, and other clinicians is a crucial but largely missing link.

## Data Availability

No new data were created for this study.
